# Women Commencing Anastrozole, Letrozole or Tamoxifen for Early Breast Cancer: The Impact of Comorbidity and Demographics on Initial Choice

**DOI:** 10.1371/journal.pone.0084835

**Published:** 2014-01-02

**Authors:** Anna Kemp, David B. Preen, Christobel Saunders, Frances Boyle, Max Bulsara, C. D’Arcy J. Holman, Eva Malacova, Elizabeth E. Roughead

**Affiliations:** 1 Centre for Health Services Research, The University of Western Australia, Perth, Western Australia, Australia; 2 School of Surgery, The University of Western Australia, The University of Western Australia, Perth, Western Australia, Australia; 3 Patricia Richie Centre for Cancer Care and Research, The Mater Hospital, Sydney, New South Wales, Australia; 4 Institute of Health Research, University of Notre Dame, Perth, Western Australia, Australia; 5 School of Pharmacy and Medical Sciences, University of South Australia, Adelaide, South Australia, Australia; University of North Carolina School of Medicine, United States of America

## Abstract

**Background:**

Australian clinical guidelines recommend endocrine therapy for all women with hormone-dependent early breast cancer. Guidelines specify tamoxifen as first-line therapy for pre-menopausal women, and tamoxifen or an aromatase inhibitor (AI) for post-menopausal women depending on the risk of recurrence based on tumour characteristics including size. Therapies have different side effect profiles; therefore comorbidity may also influence choice. We examined comorbidity, and the clinical and demographic characteristics of women commencing different therapies.

**Patients and Methods:**

We identified the first dispensing of tamoxifen, anastrozole or letrozole for women diagnosed with invasive breast cancer in the 45 and Up Study from 2004–2009 (*N = *1266). Unit-level pharmacy and medical service claims, hospital, Cancer Registry, and self-reported data were linked to determine menopause status at diagnosis, tumour size, age, comorbidities, and change in subsidy restrictions. Chi-square tests and generalised regression models were used to compare the characteristics of women commencing different therapies.

**Results:**

Most pre-menopausal women commenced therapy with tamoxifen (91%). Anastrozole was the predominant therapy for post-menopausal women (57%), followed by tamoxifen (28%). Women with osteoporosis were less likely to commence anastrozole compared with tamoxifen (anastrozole RR = 0.7, 95% CI = 0.5–0.9). Women with arthritis were 1.6-times more likely to commence letrozole than anastrozole (95% CI = 1.1–2.1). Tamoxifen was more often initiated in women with tumours >1 cm, who were also ≥75 years. Subsidy restriction changes were associated with substantial increases in the proportion of women commencing AIs (anastrozole RR = 4.3, letrozole RR = 8.3).

**Conclusions:**

The findings indicate interplay of comorbidity and therapy choice for women with invasive breast cancer. Most post-menopausal women commenced therapy with anastrozole; however, letrozole and tamoxifen were more often initiated for women with comorbid arthritis and osteoporosis, respectively. Tamoxifen was also more common for women with tumours >1 cm and aged ≥75 years. Subsidy restrictions appear to have strongly influenced therapy choice.

## Introduction

Endocrine therapy is the foundation of post-surgical treatment for women with hormone-dependent early breast cancer [1]. Therapy acts to either block the production of oestrogen or the stimulatory action of oestrogen on tumour cells [1]. When taken for five years, endocrine therapies halve the risk of recurrence and significantly reduce cancer-related mortality [2]–[4]. Clinical trial evidence supports the use of tamoxifen, or an aromatase inhibitor (AI); anastrozole, letrozole and exemestane, as first-line endocrine therapy [2], [4], [5]. The effectiveness and tolerability of these therapies varies depending on the menopause status and clinical characteristics of the individual woman [6].

AIs act by inhibiting the production of oestrogen in non-ovarian tissue and are, therefore, only suitable for women whose ovarian production of oestrogen has ceased (i.e. they are post-menopausal) [1], [3]. Anastrozole and letrozole have superior disease-free survival to tamoxifen in post-menopausal women [5], [7], [8]; however, their use is associated with musculoskeletal pain, decreased bone density, increased risk of fractures, and cardiovascular disease [2], [5], [9], [10]. Clinicians or patients may be hesitant to use AIs where there is a history of arthritis, osteoporosis, atrial fibrillation or myocardial infarction. Tamoxifen is suitable for use in either pre- or post-menopausal women [3]. Tamoxifen may be better tolerated by elderly women [8] but is associated with an elevated risk of endometrial complications including cancer, and thromboembolic events [1], [4]. Clinicians and patients hay be hesitant to commence tamoxifen, therefore, where there is a history of endometrial cancer, deep vein thrombosis (DVT), pulmonary embolism (PE) or stroke.

Tamoxifen, anastrozole and letrozole are the publically-subsidised first-line therapies for treatment of hormone-dependent early breast cancer in Australia [11]. Current Australian clinical guidelines recommend post-surgical endocrine therapy for all women with hormone-dependent early breast cancer, and indicate specific therapies for post-menopausal women depending on recurrence risk, which is defined according to tumour characteristics including size [12] (Table 1). Guidelines indicate that therapy choice should be based on the likely risks and benefits for each individual but guidelines are not specific in relation to comorbidity [12], [13].

**Table 1:** Australian clinical guidelines for endocrine therapy use in women with hormone-dependent early breast cancer [12], [13].

Menopause status	Treatment guidelines
Pre-menopausal	Tamoxifen only
Post-menopausal	Choice of endocrine therapy based on risk of recurrence
	*High risk (tumours >2* *cm)*
	• Endocrine therapy should commence with an aromatase inhibitor
	*Intermediate risk (tumours 1.1–2* *cm)*
	• Adjuvant endocrine therapy with an aromatase inhibitor is recommended
	*Low risk (tumours ≤1* *cm)*
	• Treatment with tamoxifen is recommended rather than with an aromatase inhibitor as the balance between benefits and harms of aromatase inhibitor is unclear for such women

It is not known whether prescribing of endocrine therapies in Australian practice is consistent with current guidelines in terms of menopause status and recurrence risk. It is unclear whether comorbidity is considered when selecting an endocrine therapy, or which therapy is chosen in instances where menopause status, recurrence risk or comorbidity alone point to different therapies. The aim of this study was to determine the clinical and demographic characteristics of women commencing different endocrine therapies for early breast cancer in Australian practice.

## Methods

### Ethics Statement

Participants were drawn from the 45 and Up Study cohort. All cohort members provided written consent to join 45 and Up Study, have their routinely-collected health data linked, and for these data to be provided to third-party researchers for approved projects. This consent procedure was approved by the University of New South Wales Human Research Ethics Committee and the Australian Government Department of Health and Ageing. The current study also received approval from The University of Western Australia Human Research Ethics Committee (approval RA/4/1/4589) and the NSW Population and Health Services Research Ethics Committee (approval HREC/11/CIPHS/35).

### Study Sample

Cases were selected from participants enrolled in the 45 and Up Study; a cohort of approximately 267,000 adults (143,014 women) aged ≥45 years residing in New South Wales (NSW), Australia [14]. Participants joined the study between January 2006-April 2009 and completed a baseline questionnaire of demographic and health-related items [15]. Baseline data were linked to pharmaceutical and medical service claims subsidised by the Australian government and other datasets can be linked on a project-by-project basis. Information regarding the establishment and recruitment of the cohort are described elsewhere [14].

### Data Sources and Linkage

We accessed unit-record, linked data from: i) the 45 and Up Study baseline survey, ii) NSW Cancer Registry, iii) NSW Admitted Patient Data Collection, iv) Pharmaceutical Benefits Scheme (PBS) claims, and v) Medicare Benefits Schedule (MBS) claims. Linkage of PBS and MBS data were conducted by the Department of Human Services, and remaining datasets by the NSW Centre for Health Record Linkage [16]. Researchers were provided with de-identified data only. The study period was defined as 1 January 2003 to 30 June 2011.

### Inclusion Criteria

Cases included in the study were women with a diagnosis of invasive breast cancer between January 2004-December 2009, ascertained through the: i) NSW Cancer Registry and ii) Admitted Patient Data Collection. The Cancer Registry contains, by statutory requirement, records of all cancers diagnosed or treated in NSW [17], [18] and is considered the ‘gold standard’ for cancer identification for research purposes [18]. Women with a diagnosis of invasive breast cancer listed on the Cancer Registry between January 2004-December 2008 were included in the study (*N* = 2240). International Classification of Diseases version 10 with Australian modifications (ICD-10-AM) codes C50.0-C50.9 were used to identify cases [19]. The month and year of diagnosis recorded on the Cancer Registry were considered the diagnosis date. The NSW Cancer Registry has not released incidence data since the end of 2008 [20]. Therefore, cases of invasive breast cancer diagnosed in 2009 were identified by inpatient primary diagnosis of invasive breast cancer with the same ICD-10-AM codes (*N* = 470). The first admission with this diagnosis for these women was considered the diagnosis date. Hospital-derived diagnosis had a positive predictive value and sensitivity of 86%, and specificity of 99.9%, compared with the Cancer Registry [20], [21]. Sensitivity analyses showed that 53% of women with a Cancer Registry diagnosis had an inpatient diagnosis within the same calendar month, and 93% within one calendar month.

### Identifying the Initial Endocrine Therapy

We identified claims for dispensing of prescribed medicines listed on the PBS. The PBS entitles all Australian citizens and permanent residents to subsidised medicines [22]. Initial therapy was defined as the first PBS record for tamoxifen (PBS items 2109B, 2110C), anastrozole (8179L) or letrozole (8245Y) within 18 months of follow-up from diagnosis. We did not include exemestane as it was restricted to advanced breast cancer [23]. The 18-month follow-up period was chosen based on evidence that endocrine therapy is initiated within this timeframe [21], [24]. Of the 1832 women in our dataset dispensed an endocrine therapy during the study period, sensitivity analysis showed that 96% (*N* = 1763) had been dispensed a therapy within 18 months of diagnosis.

### Exclusion Criteria

Women with metastatic cancer at diagnosis (*N = *47) or whose menopause status at diagnosis could not be ascertained (*N* = 425, described below) were excluded. Individuals were also excluded if they did not have an endocrine therapy dispensed within 18 months of diagnosis (*N = *947) or were dispensed an endocrine therapy before diagnosis (*N = *25). The final study sample comprised 1266 women.

### Comorbidities

Comorbid osteoarthritis or rheumatoid arthritis (‘arthritis’), atrial fibrillation, DVT or PE, endometrial cancer, myocardial infarction (MI), osteoporosis, and stroke were ascertained as history of these conditions may influence therapy choice. Comorbidity was defined by: i) dispensing of ≥1 specified PBS items prior to diagnosis, or ii) a hospital admission with specified ICD-10-AM codes in the primary or other diagnosis field prior to diagnosis (Table 2). Medicines for DVT and PE are not specific to these conditions; therefore, these comorbidities were identified by ≥1 enoxaparin, heparin or warfarin dispensing, with an MBS claim for venous Doppler ultrasound, computerised tomography pulmonary angiography, D-dimer blood test, or a ventilation perfusion lung scan prior to diagnosis.

**Table 2:** Diagnosis, pharmacy and procedure codes used to identify history of specified conditions.

Comorbidity	ICD-10-AM^a^	Generic name and PBS^b^ item code	Subsidy restrictions
Arthritis(osteoarthritis andrheumatoidarthritis)	M05*–*M06,M08.0, M12,M15*–*M19,M45.	Celecoxib (8439E, 8440F), diclofenac(1299J, 1300K), ibuprofen (3190X),indomethacin (2454E); ketoprofen(1590Q); meloxicam (8561N, 8562P,8887R, 8888T); naproxen (1614Y,1615B, 1659H, 1674D, 1795L);piroxicam (1895R, 1896T, 1897W,1898X); rofecoxib (8471W, 8472X);sulindac (2048T, 9032J); tiaprofenicacid (2103Q).	Severe arthropathies withan inflammatorycomponent includingosteoporosis, or bonepain due to malignantdisease, or symptomatictreatment of rheumatoidarthritis
Atrialfibrillation	I48.0*–*I48.9	*–*	*–*
Deep veinthrombosis orpulmonaryembolism	I26.0, I26.8,I26.8, I80.0*–*I80.9.	Enoxaparin (8262W, 8263X, 8264Y,8510X, 8558K, 9195Y); heparin(1076P, 1463B, 1466E);warfarin (2843P, 2209G, 2211J).	Doppler ultrasoundof veins (11602^c^, 55244),scan for venous disease(11604), scan of lowerlimb veins (11605),computerisedtomography pulmonaryangiography (57350,57351, 57356), D*–*dimerblood test (65120,65123, 65126, 65129),ventilation perfusionlung scan (61348).
Endometrialcancer	C54.1	*–*	*–*
Myocardialinfarction	I21.0*–*I22.9	*–*	*–*
Osteoporosis	M80*–*M82	Alendronate (8102K, 8511Y, 9012H,9183H, 9351E); etidronate (8056B);raloxifene (8363E); risedronate (8481J,8621R, 8899J, 9147K, 9391G);strontium (3036T); zoledronate(9288W).	Severe osteoporosis(fracture sustained or a Tscore <3).
Stroke	I63.0*–*I63.9	*–*	*–*

### Ascertaining Menopause Status

Menopause status at diagnosis was derived from: i) age at diagnosis, ii) self-reported menopause status provided in the 45 and Up Study baseline survey, or iii) whether the baseline survey was completed before or after diagnosis. Women were classified as post-menopausal if they were aged ≥55 years at diagnosis [6], or if they were <55 years and reported being post-menopausal before diagnosis. Women aged <55 years at diagnosis and reporting being pre-menopausal after diagnosis were classified as pre-menopausal. The rest of the women aged <55 who reported: i) being pre-menopausal before diagnosis, ii) post-menopausal after diagnosis, or iii) unknown menopause status were classified as having ‘unknown menopause status’, since their status could have changed between time of reporting and diagnosis.

### Other Covariates

Tumour stage and size were listed in the Cancer Registry. Stage data were provided as ‘localised’, ‘regionalised’, ‘distant’, or ‘unknown or missing’. Tumour size was categorised as ‘≤1 cm’, ‘>1–2 cm’, ‘>2 cm’, or ‘unknown or missing’. Residential location was assessed using the Accessibility/Remoteness Index of Australia Plus (ARIA+) [25]. Area disadvantage was assessed using the Socio-Economic Indexes for Areas [26] divided into tertiles.

Prior to December 2005, anastrozole was only PBS-subsidised for advanced breast cancer or for early breast cancer where tamoxifen was contra-indicated or not tolerated [27]. Before December 2006 letrozole was restricted for treatment of advanced breast cancer only [28]. After these respective dates clinicians could prescribe anastrozole or letrozole for any hormone-dependent breast cancer in post-menopausal women. Therefore, we included a term to represent therapies dispensed before or after the subsidy restriction changes (before/after December 2005 and before/after December 2006).

The PBS dataset did not capture dispensing to ‘general’ beneficiaries for medicines priced below the co-payment during the study period (A$33.30 in 2010) [29]. Most of the medicines examined were priced above this co-payment and were therefore captured. However, all the arthritis medicines, warfarin and some heparin cost less than the co-payment and were only captured for the subset of participants receiving social security (62%).

### Statistical Analyses

Chi-square and analysis of variance tests were used to compare the characteristics of women initially dispensed tamoxifen, anastrozole or letrozole. Further chi-square tests were conducted to compare the proportion of women with specified characteristics dispensed different therapies, stratified by tumour size. Multivariate modified Poisson regression models were conducted to determine the associations between patient characteristics and dispensing of different therapies. The dependent variables for these models were binary: the number of women commencing therapy with; i) anastrozole compared with tamoxifen, ii) letrozole compared with tamoxifen, and iii) letrozole compared with anastrozole. A modified regression approach was selected over logistic regression because the outcomes (numbers of women commencing specific therapies) were not rare [30].

All variables which varied by therapy type in the previous analyses (*P*≤0.05) were included in the multivariate models. Adjusted rate ratios (RR) and 95% confidence intervals (CIs) were calculated for each variable. Sensitivity analyses were conducted to determine the impact on the multivariate analyses of: i) changing the menopause age cut-off from 55 to 58 years and ii) removing the 2009 cases identified through hospital records. Changing the menopause cut-off did not alter the results of the multivariate analyses (results not shown). Removing the 2009 cases did not influence the RR but resulted in wider CIs for comorbidity variables due to reduced power (results not shown). Therefore, we retained the menopause cut-off at 55 years and included the 2009 cases in the final analyses. All analyses were conducted using IBM SPSS version 19.

## Results

### Unadjusted Analyses

Of the 1266 women included in the final sample, 55 were pre-menopausal at diagnosis and 1211 were post-menopausal. The majority of pre-menopausal women (91%) commenced therapy with tamoxifen. Only five pre-menopausal women were initiated on anastrozole or letrozole. Choice of initial therapy for post-menopausal women changed substantially after subsidy restriction changes. Therefore, the unadjusted comparisons of patient characteristics and initial therapy were restricted to women commencing therapy from December 2005 (*N* = 919).

Anastrozole was the most commonly initiated therapy for post-menopausal women (57%), followed by tamoxifen (28%) (Table 3). Choice of therapy varied significantly by age, tumour size and stage. Women commencing tamoxifen were more likely to be ≥75 years, have tumours ≤1 cm and have localised tumours compared with women commencing anastrozole or letrozole. Residential location, disadvantage, and birth country did not vary by initial therapy. Women initiated on anastrozole were more likely to have regionalised tumours compared with those commencing other therapies. Tamoxifen was initiated more often than other therapies for women with osteoporosis, and letrozole more often in women with arthritis. Therapy type did not vary by history of atrial fibrillation, DVT or PE, endometrial cancer, MI, or stroke.

**Table 3:** Characteristics of post-menopausal women commencing therapy with tamoxifen, anastrozole or letrozole between December 2005 and December 2010.

Patient and clinical characteristics	Tamoxifen	Anastrozole	Letrozole	Total *N*	*P*-value
*N*	255 (27.7%)	521 (56.7%)	143 (15.6%)	919	*–*
Mean age in years (sd)	67.8 (9.7)	65.4 (7.6)	66.4 (7.3)	919	0.001
Age:					
<55 years	4 (1.6%)	10 (1.9%)	4 (2.8%)	18	0.005
55*–*74 years	194 (76.1%)	448 (86.0%)	119 (83.2%)	761	
≥75 years	57 (22.3%)	63 (12.1%)	20 (14.0%)	140	
Tumour size:					
≤1 cm	57 (22.4%)	58 (11.1%)	14 (9.8%)	129	<0.001
<1*–*2 cm	83 (32.5%)	175 (33.6%)	37 (25.9%)	295	
>2 cm	53 (20.8%)	140 (26.9%)	30 (21.0%)	223	
Missing	62 (24.3%)	148 (28.4%)	62 (43.4%)	272	
Stage:					
Localised	133 (52.2%)	207 (39.7%)	44 (30.8%)	384	<0.001
Regionalised	65 (25.5%)	177 (34.0%)	38 (26.6%)	280	
Missing	57 (22.4%)	137 (26.3%)	61 (42.7%)	255	
Comorbidities:					
Arthritis	92 (36.1%)	157 (30.1%)	65 (45.5%)	314	0.002
Atrial fibrillation	9 (3.5%)	13 (2.5%)	5 (3.5%)	27	0.661
DVT or PE^a^	11 (4.3%)	22 (4.2%)	10 (7.0%)	43	0.361
Endometrial cancer	0 (0.0%)	3 (0.6%)	0 (0.0%)	3	0.317
Myocardial infarction	2 (0.8%)	7 (1.3%)	1 (0.7%)	10	0.692
Osteoporosis	51 (20.0%)	44 (8.4%)	15 (10.5%)	110	<0.001
Stroke	0 (0.0%)	2 (0.4%)	1 (0.7%)	3	0.473
Location:					
Major city	118 (46.3%)	221 (42.4%)	55 (38.5%)	394	0.176
Regional	85 (33.3%)	209 (40.1%)	65 (45.5%)	359	
Remote	52 (20.4%)	91 (17.5%)	23 (16.1%)	166	
Disadvantage:					
1 (most)	87 (34.1%)	170 (32.6%)	56 (39.2%)	313	0.213
2	75 (29.4%)	188 (36.1%)	43 (30.1%)	306	
3 (least)	93 (36.5%)	163 (31.3%)	44 (30.8%)	300	
Birth country:					
Australia	181 (71.0%)	385 (73.9%)	109 (76.2%)	675	0.717
UK, NZ^b^	41 (16.1%)	70 (13.4%)	16 (11.2%)	127	
Other	33 (12.9%)	66 (12.7%)	18 (12.6%)	117	

^a^ Deep vein thrombosis or pulmonary embolism.

^b^ United Kingdom and New Zealand.

### Characteristics of Women Initiated with Different Therapies Stratified by Tumour Size

**Figure 1:**
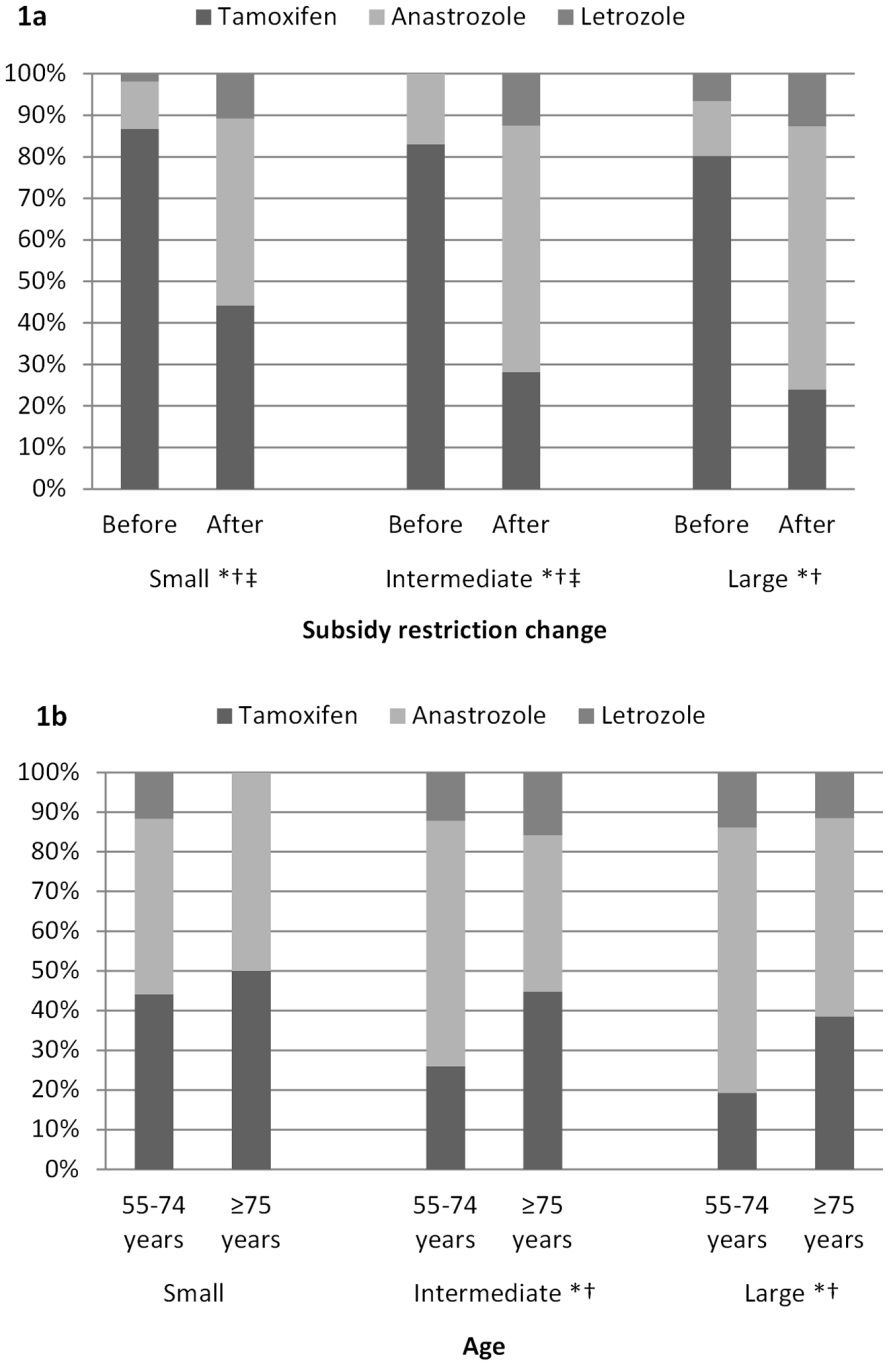
a–b: Proportion of post-menopausal women commencing different endocrine therapies, by specified characteristics, and stratified for tumour size. 1a includes women commencing therapy between January 2004-December 2010. 1b is restricted to women commencing therapy between December 2005-December 2010 to reflect practice after the subsidy restriction changes. *Difference of P<0.05 in the proportion of women commencing tamoxifen. ^†^Difference of P<0.05 in the proportion of women commencing anastrozole. ^‡^ Difference of P<0.05 in the proportion of women commencing letrozole.

The impact of the change to subsidy restrictions was observed for women with tumours of all sizes (Figure 1a). Age significantly impacted on therapy type for women with intermediate (>1–2 cm) or large (>2 cm) tumours (Figure 1b). Women aged ≥75 years and with intermediate or large tumours were more likely to commence tamoxifen than women 55–74 years (intermediate 45% vs. 26%, *P = *0.017; large 38% vs. 19%, *P = *0.009), and were less likely to commence anastrozole (intermediate 39% vs. 62%, *P = *0.005; large 50% vs. 67%, *P = *0.028).

### Adjusted Analyses

Multivariate analyses showed that women were 4-times more likely to commence treatment with anastrozole than tamoxifen following the restriction changes (95% CI = 3.1–5.9), and more than 8-times more likely to commence with letrozole (95% CI = 4.1–16.5) (Table 4). Age did not impact on therapy choice after adjustment for other covariates. Women with large tumours were significantly more likely to commence therapy with an AI than tamoxifen (anastrozole vs. tamoxifen RR = 1.4, 95% CI = 1.0–1.9, *P* = 0.039; letrozole vs. tamoxifen RR = 2.0, 95% CI = 1.1–3.8, *P* = 0.030). Women with regionalised tumours were also significantly more likely to be initiated on anastrozole than tamoxifen (RR = 1.3, 95% CI = 1.0–1.5, *P* = 0.028).

**Table 4:** Results from multivariate Poisson regression models: impact of clinical and demographic characteristics on commencing different endocrine therapies between January 2004 and December 2010 for post-menopausal women with invasive breast cancer.

Patient and clinical characteristics	Anastrozole vs. tamoxifen		Letrozole vs. tamoxifen		Letrozole vs. anastrozole	
	Rate ratio	*P*-value	Rate ratio	*P*-value	Rate ratio	*P*-value
Tumour size at diagnosis:						
≤1 cm	*–*	*–*	*–*	*–*	*–*	*–*
>1*–*2 cm	1.32 (0.99*–*1.76)	0.054	1.52 (0.83*–*2.79)	0.176	0.85 (0.46*–*1.53)	0.591
>2 cm	1.37 (1.02*–*1.85)	0.039	2.01 (1.07*–*3.76)	0.030	0.99 (0.63*–*1.85)	0.969
Missing	1.32 (0.82*–*2.15)	0.254	1.43 (0.57*–*3.60)	0.452	0.76 (0.23*–*2.53)	0.652
Tumour stage at diagnosis:						
Localised	*–*	*–*	*–*	*–*	*–*	*–*
Regionalised	1.25 (1.03*–*1.52)	0.028	1.30 (0.84*–*2.00)	0.234	0.91 (0.59*–*1.42)	0.687
Missing	1.16 (0.74*–*1.83)	0.515	2.18 (0.92*–*5.19)	0.077	2.04 (0.67*–*6.22)	0.212
Age:						
<55 years	*–*	*–*	*–*	*–*	*–*	*–*
55*–*74 years	1.02 (0.54*–*1.92)	0.948	1.04 (0.38*–*2.78)	0.936	0.72 (0.26*–*1.95)	0.515
≥75 years	0.79 (0.41*–*1.55)	0.499	0.78 (0.26*–*2.32)	0.652	0.81 (0.28*–*2.39)	0.705
After subsidy restriction change	4.30 (3.13*–*5.89)	<0.001	8.25 (4.13*–*16.5)	<0.001	*–*	*–*
Comorbidities:						
Arthritis	0.98 (0.81*–*1.18)	0.828	1.26 (0.90*–*1.76)	0.182	1.55 (1.12*–*2.14)	0.009
Osteoporosis	0.69 (0.51*–*0.94)	0.020	0.59 (0.34*–*1.01)	0.055	1.14 (0.66*–*1.95)	0.646

Comorbidity was a predictor of therapy type after adjustment for other covariates. Women with osteoporosis were 31% less likely to be initiated on anastrozole than tamoxifen (RR = 0.7, 95% CI = 0.5–0.9, *P* = 0.020), and 41% less likely to commence letrozole compared with tamoxifen (RR = 0.6, 95% CI = 0.3–1.0, *P* = 0.055). Post-menopausal women with arthritis were 1.6-times more likely to be initiated on letrozole compared with anastrozole (95% CI = 1.1–2.1, *P* = 0.009).

## Discussion

Clinical guidelines recommend particular therapies based on tumour characteristics including size, and choice may be influenced by comorbidity; but women can have both. The majority of post-menopausal women were initiated on anastrozole or letrozole; however there was evidence of comorbidity being considered when selecting initial therapy. Women with osteoporosis were significantly more likely to commence with tamoxifen compared to women without osteoporosis. Contrary to what might have been expected, women with arthritis were not more likely to commence therapy with tamoxifen but were significantly more likely to begin with letrozole. Recent evidence suggests that letrozole may be better tolerated by women with arthritis than anastrozole [31], [32], and clinicians may have considered this AI a better alternative to anastrozole than tamoxifen given its superior efficacy [8], [33]. Trade-offs were apparent for women ≥75 years with intermediate or large tumours, who were more likely to commence therapy with tamoxifen than their younger counterparts. Tamoxifen is often better tolerated by the elderly [8], and clinicians may be trading-off these benefits over the possibility of decreased disease-free survival in this group.

Our findings suggest that pharmaceutical insurance policies have a large impact on therapy choice for women with early breast cancer. Restriction changes in 2005 and 2006 allowed public-subsidy of anastrozole and letrozole for all post-menopausal women with hormone-dependent tumours [27], [28]. These subsidy changes effectively reduced the monthly out-of-pocket cost of AIs from approximately $150 to <$35 [23] and increased the likelihood of commencing an AI by between 4- and 8-times. This shift represents appropriate prescribing in post-menopausal women with intermediate and large tumours. Current guidelines do not recommend AIs for women with tumours ≤1 cm given the unclear balance of risks and benefits in this group [13].

Consistent with current clinical guidelines the vast majority of pre-menopausal women in this study (91%) commenced therapy with tamoxifen [12]. Only five pre-menopausal women commenced therapy with an AI. Further investigation indicated that these women had received neo-adjuvant chemotherapy, which may have led to iatrogenic menopause. This may have been the reason they commenced on an AI; alternatively, these women may represent a minority receiving inappropriate prescribing.

Demographic characteristics such as area disadvantage, residential remoteness and being born outside Australia did not impact on therapy type. We found no evidence to indicate that prior atrial fibrillation, DVT, PE, MI or stroke were considered when selecting therapy; however we were underpowered for these comparisons.

### Strengths and Weaknesses

To our knowledge, this is the first study to examine the factors associated with incident dispensing of endocrine therapies to women with invasive breast cancer; either in Australia or elsewhere. Consequently, this study provides the first evidence of the clinical practice of endocrine therapy for early breast cancer outside trial conditions. We have used health and medical records for a heterogeneous community sample for whom all publically-subsidised endocrine therapies have been captured.

Some limitations exist which may have implications for this study. Our sample was drawn from the 45 and Up Study, limiting the sample to individuals ≥45 years and consenting to linkage of their health records. The health service history of such individuals may differ from younger people, or those do not agree to participate in cohort studies. Although we could not directly ascertain whether participants had hormone-dependent tumours, endocrine therapies in Australia are only publically-subsidised for hormone-dependent tumours [11]. Only women without hormone-dependant tumours or using therapies for primary prevention would be ineligible for publically-subsidised therapy [11].

We were limited to analysing women who commenced therapy and could not determine how many women were never prescribed endocrine therapy, or did not fill an initial prescription. We were not able to assess the role of patient preference in selection of therapy. Comorbidity was identified through hospital records and dispensing of medicines, with up to eight years of data available depending on diagnosis date. Consequently, we may have misclassified participants not receiving hospital or pharmaceutical treatment. Under-ascertainment of some medicines may have occurred for participants without social security status during the study period.

### Conclusion

The findings indicate that there is interplay of comorbidity and choice of therapy for women with invasive breast cancer. Most post-menopausal women commenced therapy with anastrozole; however, letrozole and tamoxifen were more likely to be initiated for women with arthritis and osteoporosis, respectively. Tamoxifen was also more commonly initiated for women with intermediate or large tumours and aged ≥75 years. The findings also suggest the changes in subsidy restrictions had a substantial impact on prescribing of endocrine therapies in Australia.
